# Challenges in Implementing Personalized Medicine for Lung Cancer within a National Healthcare System

**DOI:** 10.3390/jpm2030077

**Published:** 2012-09-10

**Authors:** David E. Dawe, Peter M. Ellis

**Affiliations:** 1 University of Manitoba, 820 Sherbrook Street, Winnipeg, MB R3A 1R8, Canada; E-Mail: david.dawe@cancercare.mb.ca; 2 Department of Medical Oncology, McMaster University, Hamilton, ON, L8S 4L8, Canada; 3 Juravinski Cancer Centre, 699 Concession Street, Hamilton, ON, L8V 5C2, Canada

**Keywords:** personalized medicine, non-small cell lung cancer, delivery of healthcare, molecular targeted therapy, erlotinib, gefitinib, crizotinib, epidermal growth factor receptor

## Abstract

The traditional approach to the treatment of advanced non-small cell lung cancer (NSCLC) relied on the uniform use of cytotoxic chemotherapy. Over the last eight years, this paradigm of care has been shifting towards the use of molecularly targeted agents. Epidermal growth factor receptor (*EGFR*) mutations have emerged as an important biomarker for these targeted agents and multiple studies have shown that tyrosine kinase inhibitors (TKI) that inhibit EGFR are superior to traditional chemotherapy in patients possessing an *EGFR* mutation. Nationally funded health care systems face a number of challenges in implementing these targeted therapies, most related to the need to test for biomarkers that predict likelihood of benefiting from the drug. These obstacles include the challenge of getting a large enough tissue sample, workload of involved specialists, reliability of subtyping in NSCLC, differences in biomarker tests, and the disconnect between the funding of drugs and the related biomarker test. In order to improve patient outcomes, in a national healthcare system, there is a need for governments to accept the changing paradigm, invest in technology and build capacity for molecular testing to facilitate the implementation of improved patient care.

## 1. Introduction

The last two decades have seen a significant shift in the approach to the management of non-small cell lung cancer (NSCLC). Prior to publication of the Non Small Cell Lung Cancer Trialists overview in 1995 [1], there was significant nihilism about the role of systemic therapy in the treatment of advanced NSCLC. Since that time multiple randomized trials have demonstrated that first-line chemotherapy with platinum-based doublets offers modest improvements in survival from 4–5 months to 8–10 months [2,3]. Additionally, trials demonstrate a benefit from second line chemotherapy [4] and maintenance therapy [5,6], further extending expected survival. Nevertheless, we seem to have reached a plateau regarding the benefit of cytotoxic chemotherapy. Within the last eight years, a better understanding of lung cancer biology together with evidence of benefit for new molecularly targeted therapies has resulted in a shift towards more individualized treatment decisions for patients with advanced NSCLC. This article examines the contrast between traditional approaches to the treatment of NSCLC and personalized treatment approaches and highlights the challenges of implementing personalized treatment approaches for patients with advanced NSCLC. 

## 2. Traditional Approach to the Treatment of NSCLC

Traditionally, all NSCLC histologies have been considered comparable enough that they were treated in a similar fashion. Older trials demonstrated that platinum-based chemotherapy improved overall survival in comparison to best supportive care (BSC) [1]. Modest gains in survival were observed in trials evaluating newer agents such as paclitaxel, vinorelbine or gemcitabine in combination with a platinum compound, although these agents all appeared to be similarly effective [3,7]. During the last decade, evidence emerged supporting a number of systemic treatment options for second-line therapy of NSCLC. Docetaxel was initially shown to improve survival in comparison to BSC [4] and subsequently pemetrexed was proven to be non-inferior to docetaxel [8]. More recently, second or third-line therapy with erlotinib, a tyrosine kinase inhibitor (TKI) of the epidermal growth factor receptor (EGFR), was shown to improve survival in patients not suitable for further chemotherapy [9]. 

These agents all found their way into routine treatment algorithms of patients with advanced NSCLC [10,11]. However, the observed benefits were modest and it was clear that not all patients derived benefit from treatment. As such, physicians used clinical characteristics such as disease stage, weight loss, co-morbidity, performance status, and age to help in patient selection for treatment [12,13,14]. Guidelines from the American Society of Clinical Oncology (ASCO) suggested the use of single-agent rather than doublet chemotherapy in elderly patients or those with poor performance status [15]. These recommendations though, have been put into question because of recent data showing that the elderly derive a similar benefit from treatment with a platinum doublet [16]. 

It is clear that this traditional approach of treating all patients with advanced NSCLC in the same manner has limitations. The emerging knowledge about molecular pathways important in the development and growth of NSCLC, as well as the identification of molecularly targeted therapies has highlighted the need for better mechanisms to identify which patients will benefit from treatments and to avoid exposing other patients to unnecessary toxicities. 

## 3. Personalized Approaches to the Treatment of NSCLC

Personalizing treatment is not a new concept. As discussed above, physicians routinely use patient and clinical characteristics to provide an individual patient with the greatest chance of benefit and least likelihood of harm. However, the National Cancer Institute defines personalized medicine as “A form of medicine that uses information about a person’s genes, proteins, and environment to prevent, diagnose, and treat disease” [17]. This definition has become increasingly important because it provides a conceptual framework in which to discuss new targeted therapies that require both careful patient selection and the need for biomarkers to guide selection of therapy. 

### 3.1. The Impact of Histology on Treatment Decisions

Trials have shown that many chemotherapeutic regimens work equally well [3]. Platinum-doublets in combination with paclitaxel, docetaxel, vinorelbine or gemcitabine all demonstrate similar response rates, time to progression and overall survival. Retrospective analyses of data from multiple NSCLC clinical trials performed by the Southwest Oncology Group (SWOG) demonstrated no differential effect of histology on either progression free or overall survival [18]. As a result histology was not routinely used in older treatment algorithms.

More recent data, using newer systemic therapies, have convincingly demonstrated that histology is predictive of either benefit or toxicity from treatment and that accurate histological subtyping should be performed for all NSCLC specimens [19]. A retrospective analysis of the JMEI trial of second-line chemotherapy with docetaxel or pemetrexed demonstrated a differential effect from histology. Patients with non-squamous histology treated with pemetrexed had improved overall survival compared to patients receiving docetaxel (hazard ratio (HR) 0.82 *vs.* 1.40, interaction test (int) p = 0.04). The opposite effect was observed for patients with squamous histology [20]. A prospective analysis of the first-line trial comparing cisplatin plus gemcitabine (a routinely used standard) with cisplatin plus pemetrexed showed a similar predictive effect from histology [21]. The combination of cisplatin-pemetrexed resulted in superior survival in patients with adenocarcinoma or large cell carcinoma (HR 0.84 *vs.* 1.23, int p = 0.002), but that cisplatin-gemcitabine was superior in patients with squamous cell carcinoma (SCC) [21]. These findings are supported by *in vitro* data examining the expression of thymidylate synthase (TS), the principal target of pemetrexed [22]. Ceppi *et al*. examined TS expression in resected NSCLC samples [22]. They found higher TS expression in squamous cancers suggesting a mechanism for the relative resistance of squamous cancers to pemetrexed.

Interestingly, pemetrexed is not the only systemic therapy for NSCLC that has shown a differential effect on outcomes. The development of bevacizumab, a monoclonal antibody targeting the vascular endothelial growth factor receptor (VEGFR), was limited to patients with non-squamous histology. In a randomized phase II trial, patients with SCC treated with bevacizumab experienced an increased risk of life threatening hemoptysis that was not seen in other histologies [23]. This led to the exclusion of bevacizumab from treatment algorithms for NSCLC patients with SCC histology. Analysis of the subsequent randomized phase III trial of carboplatin, paclitaxel with or without bevacizumab demonstrated that patients with adenocarcinoma had the greatest survival benefit from bevacizumab [24].

### 3.2. Molecular Profiling of NSCLC

The reasons why histologic subtypes of NSCLC may differ in prognosis and response to treatment may lie in fundamental differences in the molecular profiles that drive their respective proliferation. Adenocarcinoma and SCC are the best understood subtypes since they are the most common. Results of detailed molecular analysis of adenocarcinomas show evidence of driver mutations in up to 60% of samples. The most common abnormality in adenocarcinomas is mutation of the Kirsten rat sarcoma gene (*KRAS*), found in 25% of samples. Driver mutations with existing therapies include mutations of *EGFR* in 23% and translocations of the anaplastic lymphoma kinase (*ALK*) gene in 6% [25]. Recently, rearrangement of the c-ros oncogene 1 (*ROS1*) gene has also been shown to be a driver mutation in 1%–2% of NSCLC patients [26]. These mutations all appear to occur in a mutually exclusive manner and the actionable mutations appear more often in younger patients and never smokers [27].

In contrast to adenocarcinoma, *EGFR* mutations and *ALK* translocations do not appear to occur in SCC of the lung [28]. Molecular abnormalities in SCC involve amplification of the fibroblast growth factor receptor 1 (*FGFR1*) gene in up to 22% of patients and occasional mutations in the phosphoinositide-3-kinase catalytic alpha polypeptide (*PIK3CA*) gene and v-akt murine thymoma viral oncogene homolog 1 (*AKT1*) gene [28,29]. Targeted therapies for these molecular abnormalities are under evaluation.

### 3.3. Treatment Directed by KRAS Mutation

*KRAS* is the most common molecular abnormality in NSCLC, but to date there are no specific therapies for patients with *KRAS* mutations. *KRAS* mutations are felt to be a weak negative prognostic factor [19]. A meta-analysis of 28 NSCLC trials, including 1463 patients with adenocarcinoma, showed poorer overall survival in *KRAS* mutated patients, with a HR of 1.59 (1.26–2.02) [30]. However, uncertainty exists about the predictive value of *KRAS* mutations. *KRAS* mutations do not appear to consistently predict benefit from adjuvant chemotherapy, although recent data suggest that certain *KRAS* mutations may be associated with a lack of benefit [31]. Conflicting information exists about the ability of *KRAS* mutations to predict benefit from EGFR directed therapy. Data from the NCIC BR.21 trial showed worse survival for patients with *KRAS* mutations [32]. However, multiple other trials have failed to demonstrate any differential effect according to *KRAS* status. As such there is insufficient evidence to use *KRAS* status in NSCLC treatment algorithms [19]. 

### 3.4. EGFR Directed Therapy

The NCIC BR.21 trial of erlotinib *versus* placebo was the first trial of a molecularly targeted agent, demonstrating improved survival for NSCLC patients [9]. This trial showed a statistically significant improvement in median survival from 4.7 to 6.7 months (HR 0.70, *p* < 0.001), in an unselected population of NSCLC patients who had progressed after cytotoxic chemotherapy. Higher response rates and longer survival were observed in patients with increased EGFR protein expression, or increased *EGFR* gene copy number; however, there were no significant treatment interactions [33]. Multivariate analysis could not identify any subgroup that failed to benefit from therapy with erlotinib.

Nevertheless it was clear that certain patient subgroups-females, light/never smokers, patients with adenocarcinoma or Asian ethnicity, were more likely to respond to therapy with an EGFR TKI. In 2004, two groups separately identified activating mutations of the *EGFR* gene and their association with increased likelihood of response to EGFR TKI therapy [34,35]. Subsequent trials have reinforced that activating mutations of the *EGFR* gene are predictive of greater benefit from EGFR directed therapy [19]. 

The Iressa Pan-Asia Study (IPASS) trial randomized Asian never or light smokers with adenocarcinoma histology to carboplatin and paclitaxel chemotherapy, or gefitinib [36]. The study showed an improvement in progression-free survival (PFS) (HR 0.74, 95%CI 0.65–0.85). This study highlighted the importance of *EGFR* mutation status. Patients known to have an *EGFR* mutation had significant improvement in PFS from first-line gefitinib (HR 0.48, 95%CI 0.36–0.64). However, *EGFR* wild type patients receiving gefitinib had shorter PFS (HR 2.85, 95%CI 2.05–3.98). No significant differences were observed in overall survival, likely due to a high rate of cross over in the second line [37]. Findings from the first-SIGNAL trial lend further support to the predictive value of *EGFR* mutation status for first-line EGFR directed therapy [38]. 

Multiple trials have now shown superior PFS for first-line EGFR TKIs in *EGFR* mutation positive patients (Table 1). The first of these trials published was the West Japan Thoracic Oncology Group (WJTOG) 3405 study. This trial randomized chemotherapy naïve Japanese patients with *EGFR* mutation positive NSCLC to cisplatin plus docetaxel or gefitinib. Similar to the IPASS trial, it showed an improvement in PFS with gefitinib (HR 0.49, 95%CI 0.37–0.71), but no improvement in OS [39]. The European Randomized Trial of Tarceva *vs*. Chemotherapy (EURTAC) was the first trial to randomize patients from outside of Asia. This trial randomized patients from France, Italy, and Spain, to either erlotinib or a platinum doublet and demonstrated a similar improvement in PFS (HR 0.37, 95%CI 0.25–0.54) in favor of erlotinib [40]. Finally, the LUX-Lung 3 trial showed that a third EGFR inhibitor, afatinib, improves PFS in comparison to platinum-based chemotherapy (HR 0.58, 95%CI 0.43–0.78) [41]. 

Rates of Grade 3–5 toxicity reported in trials for the first line use of EGFR inhibitors are listed in Table 2. It is apparent that severe toxicity from EGFR inhibitor therapy is consistently less common than with cytotoxic chemotherapy and that treatment related mortality is a rare event. The main toxicities with EGFR inhibitors are rash, diarrhea, and elevation of liver enzymes, while common toxicities in those receiving cytotoxic chemotherapy are neutropenia, anemia, nausea/vomiting, fatigue, and thrombocytopenia. Discontinuation of therapy due to adverse events was also less common in patients receiving EGFR inhibitors (13% *vs*. 23%) [40]. Risk of toxicity, therefore, does not seem to be a barrier to adoption of more personalized therapy.

**Table 1:** Summary of first-line trials of Epidermal Growth Factor Receptor (EGFR) tyrosine kinase inhibitor (TKI) *versus* chemotherapy.

Trial	Treatment	Population	RR	PFS (m)	PFS (HR)	OS (m)	QoL
IPASS [36]	Gef *vs*. Cb/Pac	Mut^+^	71% *vs* . 47%		0.48	18.8 *vs*. 17.4	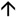
		Mut^-^	1% *vs* . 23%		2.85		
First Signal [38]	Gef *vs*. Cis/Gem	Mut^+^	85% *vs* . 37%		0.61	22.3 *vs*. 22.9	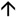
		Mut^-^	26% *vs* . 52%		1.52		
NEJ002 [42]	Gef *vs*. Cb/Pac	Mut^+^	74% *vs*. 31%	10.8 *vs*. 5.4	0.30	30.5 *vs*. 23.6	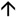
WJTOG 3405 [39]	Gef *vs*. Cis/Doc	Mut^+^	62% *vs*. 32%	9.2 *vs*. 6.3	0.49	NR	NR
Optimal [43]	Erl *vs*. Cb/Gem	Mut^+^	83% *vs*. 36%	13.1 *vs*. 4.6	0.16	NR	NR
EURTAC [40]	Erl *vs*. plt doub	Mut^+^	58% *vs*. 15%	9.7 *vs*. 5.2	0.37	19.3 *vs*. 19.5	NR
LUX-LUNG 3[41]	Afa *vs*. Cis/Pem	Mut^+^	56% *vs*. 23%	11.1 *vs*. 6.9	0.58	NR	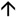

Gef–gefitinib; Erl–erlotinib; Afa–afatinib; Cb–carboplatin; Cis–cisplatin; Pac–paclitaxel; Gem–gemcitabine; Doc–docetaxel; plat doub–platinum doublet; Pem–pemetrexed; NR–not reported; QoL–quality of life; 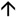
–QoL better for gefitinib; m–months; HR–hazard ratio; RR–response rate; PFS–Progression Free Survival; OS–overall survival.

**Table 2:** Summary of toxicity in first-line trials of EGFR Inhibitors.

Trial	Treatment	Overall		Grade 5	
		Grade 3-5		Toxicity	
		TKI	Chemo	TKI	Chemo
IPASS [36]	Gef *vs*. Cb/Pac	28.7%	61%	3.8%	2.7%
First Signal [38]	Gef *vs*. Cis/Gem	28.9%	68%	1.3%	0.7%
NEJ002 [42]	Gef *vs*. Cb/Pac	41.2%	71.7%	0.9%	0%
WJTOG 3405 [39]	Gef *vs*. Cis/Doc	NR	NR	1.1%	0%
Optimal [43]	Erl *vs*. Cb/Gem	17%	65%	0%	0%
EURTAC [40]	Erl *vs*. plt doub	45%	67%	1%	2%
LUX-LUNG 3 [41]	Afa *vs*. Cis/Pem	60.7%	56.8%	1.7%	0%

Gef–gefitinib; Erl–erlotinib; Afa–afatinib; Cb–carboplatin; Cis–cisplatin; Pac–paclitaxel; Gem–gemcitabine; Doc–docetaxel; plat doub–platinum doublet; Pem–pemetrexed; NR–not reported; TKI–tyrosine kinase inhibitor; Chemo–cytotoxic chemotherapy.

It is apparent from these multiple trials that the preferred first-line therapy for NSCLC patients known to have *EGFR* mutations is an EGFR TKI. The data also stress the importance of testing for the presence of mutations, rather than using clinical characteristics to predict mutation status. 

### 3.5. Other Molecularly Targeted Therapy

Two additional molecular abnormalities have been shown to predict response to a molecularly targeted agent, crizotinib. Translocations of the echinoderm microtubule-associated protein-like 4 (*EML4*)-*ALK* gene occur in 4%–7% of lung adenocarcinomas. A phase I trial of 82 patients with *EML4-ALK* translocation positive NSCLC reported a 57% objective response rate to crizotinib, with an additional 33% of patients showing stable disease [44]. Subsequent analysis of the same patients showed a 2-year overall survival of 54% [45]. Translocations of the *ROS1* gene also appear predictive of response to crizotinib [46]. These mutations occur in only 1%–2% of patients, but responses were observed in 7 of 13 patients (54%) with *ROS1* rearrangements treated with crizotinib. 

As driver mutations continue to emerge, identification of appropriate patients to receive molecularly targeted therapy can only become more important, especially when one considers the high cost of the medications.

## 4. Challenges to Implementing Personalized Treatment Approaches for the Treatment of NSCLC

The increasingly complex treatment algorithms arising as a result of molecularly driven treatments can be a challenge to implement in a nationally funded healthcare system where approval, funding, and implementation of new therapies may occur slowly. A new therapy must overcome several hurdles prior to implementation. First, there must be sufficient evidence of efficacy for the new treatment as summarized above. Secondly, toxicity must be manageable. Third, the cost-effectiveness of the new therapy must fall within the bounds acceptable by the country. Finally, one must be able to identify which patients are likely to benefit and which should not receive the novel treatment. Significant barriers to implementation may arise from these latter concepts.

### 4.1. Cost-Effectiveness

Intuitively, testing for *EGFR* mutation status improves the cost effectiveness of using EGFR TKIs in the first line by avoiding the cost of medication in patients without the appropriate molecular profile. In patients with an *EGFR* mutation, conflicting data on cost effectiveness of first-line therapy of EGFR TKIs have been reported. These findings seem to be most affected by the price level of the drug and what cost level each country considers “cost-effective”. The Pharmaceutical Benefits Advisory Committee in Australia determined that first-line therapy with gefitinib for NSCLC patients with *EGFR* mutated tumors was cost-effective, with an incremental cost effectiveness ratio (ICER) of $15,000–$45,000 per quality adjusted life year (QALY) gained [47]. Using 2010 average exchange rates, this converts to US dollar (USD) $13,227–$39,682 per QALY [48]. An analysis from the National Institute for Health and Clinical Excellence (NICE) in the United Kingdom determined an ICER of £35,700 per QALY (USD $53,046/QALY), which was not deemed to be cost effective [49]. Subsequent implementation was based on a confidential fixed price negotiated with the pharmaceutical company [50]. A similar discussion and recommendation occurred with erlotinib [51]. Finally, an Ontario Health Technology Assessment of *EGFR* testing in NSCLC patients calculated an ICER of $81,000 per QALY (USD $75,700/QALY). They stated that a decision on whether this level was cost effective depended on the province’s willingness to pay [52]. Based on the variation in the above evaluations, cost-effectiveness remains as a potential barrier to implementation of these targeted therapies, but decrease in costs of testing and drug price negotiation can help overcome this barrier.

### 4.2. Biomarker Testing and Patient Identification

Given the improved efficacy and toxicity profiles of EGFR and ALK directed therapies, physicians have been eager to implement these treatments into routine practice. However, use of these agents is limited to patient whose tumors are known to carry one of the previously described molecular abnormalities, which represents a small proportion of the NSCLC population. This highlights the need for molecular testing to identify the appropriate patients. Guidelines from the American Society of Clinical Oncology (ASCO) and a European consensus workshop now recommend testing for *EGFR* mutation in the tumor of any patient being considered for first line TKI treatment [53,54]. A Canadian consensus conference recommended routine testing for *EGFR* mutations in patients with advanced NSCLC and non-squamous histology [19]. These recommendations did not include *ALK* testing, as there was no approved therapy at that time. However, with the approval and licensing of crizotinib in the United States and Canada, *ALK* testing should also be routinely considered in patients with lung adenocarcinomas. Various challenges exist in the implementation of routine biomarker testing. 

### 4.3. Adequate Tissue Samples

A significant barrier to biomarker testing is the availability of an adequate amount of tissue due to increasing diagnostic demands and declining amounts of tissue delivered per patient [55]. Up to 80% of NSCLC patients with advanced disease will only have tissue from small biopsies or cytology [56] limiting the ability to perform additional tests. There is a strong and pressing need to improve the quality of diagnostic lung cancer samples. Thunnissen *et al*. have proposed methods for maximizing the yield of transbronchial and transthoracic biopsy, while others have reported improved sample yields with the presence of a cytopathologist at the time of biopsy [55,57]. Unfortunately, some of these measures to improve biopsy yield are not feasible to implement in smaller centers. Community hospitals may have a particularly difficult time achieving adequate tissue samples because they may lack thoracic surgeons to perform mediastinoscopy or thorascopically guided tissue sampling. To achieve an adequate sample could then require referral to a larger facility, which results in greater costs to the healthcare system, delays in treatment, and travels costs for the patient. Successfully collecting adequate amounts of tissue from all patients is a substantial obstacle to implementing standardized molecular testing for patients with NSCLC.

### 4.4. Workload of Specialists

The need for improved diagnostic lung cancer samples has major resource implications in a national healthcare system affecting thoracic surgeons, respirologists, interventional radiologists and pathologists. Limited numbers of specialists and finite operating room time make it difficult to scale up the number of procedures to collect adequate tissue. As noted above, community hospitals may also have limited access to such physicians, leading to referral. This problem is only compounded by a changing paradigm in which repeat biopsy is required at the time of disease progression to determine if there has been a change in molecular phenotype. While the data supporting re-biopsy on progression is sparse, heterogeneity in mutation profile between the primary tumor and metastases has been demonstrated [58]. Much more research is needed in this area, especially since there is a theoretical risk to patients inherent in either more extensive surgical procedures or a larger number of biopsy attempts. 

### 4.5. Reliability of Subtyping

It is clear that the molecular profiles of squamous and non-squamous cancers differ greatly. Routine molecular testing to date has been limited to patients with non-squamous histology. However, up to 40% of NSCLC cases cannot be reliably subtyped and are labeled NSCLC-NOS [59,60]. Additionally, there is poor interobserver reliability in subtyping lung cancer samples [61,62]. Routine use of immunohistochemical (IHC) testing is recommended to more accurately classify diagnostic lung cancer samples, although this requires considerable education of the pathology community and does have workload implications [56,63]. 

### 4.6. Choice of Test

Once the above issues are overcome, there remain some challenges around testing of the actual biomarker. Testing for the *EGFR* mutation is the best established of these biomarkers due to the availability of EGFR TKIs. There are at least thirteen different methodologies that can be used to test for an *EGFR* mutation, all with different costs, technical quirks, required expertise, and equipment [64]. For example, some techniques require 25% of DNA to be mutated and can only detect known mutations while others require the mutation to be present in only 0.1% of DNA and can detect known or new mutations. All are fundamentally based on either polymerase chain reaction (PCR) platforms or direct sequencing. Given the above variation in sensitivity, standardized methods for testing are needed to ensure reliability and validity of results. 

Adding to the complexity described with *EGFR* testing is that testing for biomarkers like *EML4-ALK* translocation and *ROS1* translocation requires fluorescence in-situ hybridization (FISH). While this test is now widely used, it is labor intensive and is not performed to the necessary standard at all laboratories. Also, depending on the method of *EGFR* testing being used, the two tests may require different preparation and fixation of sample tissues. When differing modes of fixation and tissue preparation are coupled with the generally small samples received by pathologists, it creates a substantial challenge to establishing efficient algorithms for *EGFR* and *EML4-ALK* testing. While the collection of larger samples will undoubtedly improve some of this difficulty, protocols must still be established to guide molecular testing and the initial handling of samples. Physicians involved in sampling tissue, performing tests, interpreting tests, and treating patients must all be aware of these algorithms to minimize the need for re-biopsy and delays in treatment.

### 4.7. Funding

Treatment paradigms for NSCLC have evolved from a model applicable to all patients, to one in which treatment decisions are individualized based upon molecular testing of lung cancer samples. The challenge to implementation of personalized decision-making in lung cancer in a nationally funded healthcare system like Canada is the need to link funding for biomarker testing with drug funding. Different groups within Health Canada are responsible for approval of new pharmaceutical therapies and diagnostic tests [65]. This disconnect between funding of a drug and its predictive biomarker test resulted in a situation where there was no mechanism for mutation testing when gefitinib was approved for use in *EGFR* mutated patients. This disconnect in funding meant that there were very few labs capable of performing the molecular test, which therefore limited access to both the test and the drug. Even if labs had been able to reliably perform the molecular test, the lack of funding serves as a significant barrier. In fact, this scenario appeared when a pan-Canadian *EGFR* mutation-testing program was initially supported with funding from AstraZeneca Canada. There was rapid uptake of testing across the country, with approximately 250 tests per month performed [66]. When this program ended after 12 months, governmental funding was not yet in place and testing rates dropped to 50–100 tests per month. The testing rates before and after this program demonstrate the importance of ensuring that approval of drugs requiring a molecular test coincides with funding of the necessary investigation. When these two decisions remain disconnected, fewer patients are able to access these exciting and potentially life prolonging therapies. As a medical community we must attempt to streamline this process by insisting that decision-makers recognize the importance of funding diagnostic tests and ensuring that we commit research resources not only to the development of new drugs, but also towards the development, validation, and dissemination of effective biomarker assays.

## 5. Future Directions

There is considerable research effort ongoing in biomarker identification of NSCLC samples. In parallel to these research efforts, clinical trial programs have evolved to rationally evaluate molecularly targeted agents. With the development of new biomarkers and next generation sequencing it is possible that we will soon be able to test each patient’s malignancy for a range of markers using a single gene array, thereby conserving tissue, decreasing cost, and achieving the ultimate goals in personalized treatment of NSCLC. In order to achieve these goals in a national healthcare system, there is a need for governments to accept the changing paradigm, invest in technology and build capacity for molecular testing to facilitate the implementation of improved patient care. 
